# Introduction of the pneumococcal conjugate vaccine in humanitarian and fragile contexts: Perspectives from stakeholders in four African countries

**DOI:** 10.1080/21645515.2024.2314828

**Published:** 2024-03-05

**Authors:** Baldeep K. Dhaliwal, Rose Weeks, Jasmine Huber, Aminata Fofana, Mohamed Bobe, Antoinette Demian Mbailamen, George Legge, Gassim Cisse, Anita Shet

**Affiliations:** a International Vaccine Access Center, Department of International Health, Johns Hopkins Bloomberg School of Public Health, Baltimore, MD, USA; b Somalia/Somaliland Country Office, Save the Children, Mogadishu, Somalia; c Programme Elargi de Vaccination, Ministère de la Santé Publique, N’Djamena, Chad; d Expanded Programme on Immunisation (EPI), National Ministry of Health, Juba, Republic of South Sudan; e Expanded Programme on Immunisation (EPI), Ministry of Health and Public Hygiene, Conakry, Republic of Guinea

**Keywords:** Pneumonia, pneumococcal conjugate vaccine, fragile contexts, health policy, decision-making

## Abstract

Childhood pneumonia causes a significant burden of preventable child morbidity and mortality in Chad, Guinea, Somalia/Somaliland, and South Sudan. Leaders from these countries have committed to reducing this burden and are preparing to introduce the pneumococcal conjugate vaccine (PCV) into their immunization programs. To support long-term sustainability for expected PCV introductions in settings afflicted by prolonged humanitarian crises this research explores national stakeholders’ perspectives on contextual factors that may influence optimal vaccine implementation. This qualitative study used purposive sampling to identify and interview stakeholders involved in vaccine decision-making. Interview transcripts were analyzed through the framework method, an approach involving charting data into pre-populated matrices. Findings from interviews with 16 key informants from government, partner organizations, and international health agencies fit within the following four overarching themes: (1) population-level vulnerabilities to pneumonia, exacerbated by climatic risks and low levels of maternal education; (2) disease burden and the interest in enhancing surveillance to monitor vaccine impact and integrate disease control efforts; (3) policy processes, including formalizing vaccine decision-making; and (4) vaccine implementation preparation, including the conduct of robust communication campaigns, training, and cold chain upgrades. This research explores perspectives from leaders in these countries which are at pivotal moments in their journeys toward introducing PCV. Widespread commitment among leaders, in addition to financial support, will facilitate vaccine introduction. Further, fostering a shared understanding among partners about context-specific determinants of program success will help build tailored implementation strategies for each country.

## Introduction

Pneumonia remains the leading infectious cause of death in children under 5, killing more than 700,000 children each year.^1^ Four countries in Africa – Chad, Guinea, Somalia/Somaliland (hereafter referred to as Somalia), and South Sudan – bear a disproportionate burden of disease. They are experiencing prolonged humanitarian crises and suffer from some of the world’s highest child mortality rates, ranging from 99 per 1,000 live births in Guinea to 122 per 1,000 live births in Somalia.^2^ Up to one-quarter of these deaths are attributable to pneumonia.^3^ A constellation of risk factors, such as malnutrition and crowding, exacerbates the risk for pneumonia among children in these four low-income countries.^4–6^ Although many countries in the region have seen substantial reductions in pneumonia mortality since 2000, mortality rates in these four countries have only decreased by 3% or less per year.^3^ This is insufficient to achieve the Sustainable Development Goal (SDG) of ending preventable child deaths by 2030.^7^

The pneumococcal conjugate vaccine (PCV) provides protection against infections caused by pneumococcus, particularly among children under 5 who are most vulnerable to pneumococcal infections.^8^ Although the World Health Organization (WHO) recommends that all countries should include PCV in their national immunization schedules, as of 2022, only 155 out of 194 WHO member states have introduced PCV into their national or subnational immunization programs.^9,10^ Of the 54 countries in Africa, only six have not yet introduced PCV into their national or subnational immunization schedules, with most African countries introducing between 2009 and 2016. Numerous studies from the region including from Kenya, Rwanda, South Africa, and Zambia have indicated that PCV introduction is associated with significant reductions in hospitalizations and deaths associated with pneumonia, sepsis, and meningitis.^11–14^ Modeling estimates project that the introduction of PCV in Chad, Guinea, Somalia, and South Sudan could prevent nearly 60,000 deaths between 2024 and 2030.^15^

The rationale for introducing PCV in these four countries is evident, and leaders across the four countries have made strong commitments to roll out the vaccine in 2024 with the help of Gavi. The next steps in vaccine introduction are typically as follows: initiate introduction planning, select a vaccine schedule and product, and decide how to proceed programmatically. This approach might include weighing options such as introducing multiple vaccines simultaneously or including catchup vaccination campaigns for children. With numerous humanitarian challenges, countries will also need to carefully evaluate their cold chain and logistics capabilities and assess sources of funding, as well as develop social mobilization plans to ensure demand generation and adequate coverage.^16^

These countries are also at varying points with other vaccine introductions, including rotavirus, human papillomavirus (HPV), and malaria vaccines. The governments of Chad, Guinea, South Sudan, and Somalia have expressed a commitment to plan to introduce rotavirus vaccine. Although Guinea is also in the planning stage for introducing HPV, the other three countries have not yet begun their introduction or planning stages. Malaria is also a priority, which South Sudan and Chad aim to introduce in the next two years. As Chad, Guinea, South Sudan, and Somalia each manage individual timelines for these introductions, it is essential to explore the factors that impact introduction and implementation activities for these vaccines.This study aimed to understand the factors that would affect introduction and implementation activities for PCV in Gavi-eligible countries with high levels of childhood pneumonia deaths, including Chad, Guinea, Somalia, and South Sudan. Although there are comprehensive agency guidelines for vaccine implementation, and valuable lessons about campaign-style vaccination in conflict settings, there remains limited evidence from the perspective of government and other implementation leaders describing constraints surrounding new vaccine introduction in challenging contexts.^17–19^ These four countries are uniquely bound by common factors such as having high childhood pneumonia burden and under-5 child mortality, facing humanitarian crises, being eligible for Gavi support, and having strong national political will for protecting children from vaccine-preventable diseases. Documenting stakeholders’ perceptions of constraints and facilitators for PCV introduction and sustainability is intended to support the planning process, introduction of PCV, and the long-term sustainability of PCV rollout.

## Methods

### Study design and description

This qualitative study used purposive sampling and semi-structured interviews of key stakeholders who are critically involved in the decision-making and rollout of PCV. In purposive sampling, the key goal is to seek the individuals with the most information about the topic, as opposed to seeking a ‘representative sample’.^20^ The key focus of our study was to elicit views of participants on factors likely to influence successful PCV introduction, rollout, uptake, and acceptance on a national scale. Tool development, data collection, and analyses were completed by the end of April 2023.

### Ethics

All participants provided informed consent orally prior to initiating their interviews. Study details and guidelines were relayed to participants before interviews began. Participants were notified that their involvement in interviews was voluntary, their identities would remain anonymous, and no compensation would be given. Additionally, participants were given the agency to decline to answer any questions that they believed were inapplicable, and they were informed that they could discontinue the interview at any time. The Institutional Review Board at the Johns Hopkins Bloomberg School of Public Health determined this key informant study to be exempt from human subjects research oversight (IRB #21009).

### Participants

The target population for our study were policymakers and leaders of implementation efforts, including immunization program managers, Ministry of Health (MoH) officials, and in-country implementing organizations. These participants were considered ‘elite’ interviewees, referring to individuals with substantial expertise in the topic of interest and typically in a position of power to create change.^21^ We used purposive sampling to initially identify participants. We initially conducted outreach to colleagues in international agencies such as World Health Organization regional offices, research organizations, and global and local Non-Governmental Organizations (NGOs). We also reached out to colleagues who had collaborated with or presently work with policy experts within the respective countries’ health ministries.

This wide outreach provided our team with connections to participants who had substantial expertise in infectious diseases, immunization programs and health policy. After conducting initial in-depth interviews with participants, we utilized snowball sampling to refer us to additional participants with similar expertise and power to facilitate change.

### Semi-structured interviews

The semi-structured interview guide included open-ended questions addressing topics such as the perceptions of disease burden and susceptibility, health priorities, decision making processes, and implementation processes, including strategies to promote vaccine uptake in the long run (Appendix 1). domains were based on previously devised frameworks^22^ designed to understand vaccine policy decision-making and develop strategies supporting evidence-informed decision-making about new vaccine adoption. Interviews were conducted virtually over video or audio-conferencing platforms and lasted between 20–60 minutes.

### Theoretical perspective: studying up

Traditionally, vaccine access research has focused its gaze ‘downstream’ by studying populations to assess their perceptions of vaccines, misinformation about vaccination services, as well as questions of vaccine acceptance and access. To gain a more well-rounded perspective, “studying up” of proximal factors has been proposed to better understand the power structures that facilitate and create the conditions that elicit population-level perceptions.^23^ When using this approach in health research, researchers explore questions by looking toward powerful health institutions, broader cultures, research, and non-governmental organizations. In this research, we chose to “study up” to limit unequal power dynamics between interviewer and interviewee, as well as to better understand conditions that facilitate decision-making and advocacy efforts.

### Data management and analysis

Participant interview recordings were transcribed using Temi (New York, NY) or Otter.ai (Mountain View, CA), were manually checked against the recording, and analyzed using the ‘framework method’, a rapid but systematic and rigorous method for the analysis of qualitative data.^24^ We initiated deductive analysis by charting data into matrices with rows for each interview guide topic. After several rounds of iterative reviews, the matrix style was finalized, and the team compiled interview excerpts into said matrices; these matrices each included a summary of participant views for each topic. By extracting data from each row of each chart, we created a summary matrix that consisted of topic summaries across all respondents. The summary matrix enabled comparisons between respondents and assessments of emerging themes and respondent concurrence and divergence. Next, data were condensed to highlight core findings for each topic. We deployed member checking, working with global and in-country technical experts to provide additional insight, which we triangulated against key informant findings, as is recommended when conducting ‘elite interviews’.^21^

## Results

A total of 16 interviews were conducted with policymakers, ministry partners, and other stakeholders across Chad, Guinea, Somalia, and South Sudan. The stakeholders involved in these interviews included MoH officials, vaccination coordinators, physicians in public service, and technical experts from WHO, Save the Children, and UNICEF. Each person interviewed was purposively selected as they held a pivotal role in immunization policy, vaccine introduction, and implementation. Details on these participants are provided in Table 1.

**Table 1.** Participant details.

Country	Interviews/number of participants	Participants’ affilations
Chad [CD]	4	NGOs, health agency, MOH
Guinea [GA]	3	MOH, health agency
Somalia [SA]	6	MOH, NGOs, Research Institution
South Sudan [SS]	3	MOH, health agency, NGO

Findings are described within four overarching themes, which are critical for fostering a successful vaccine implementation program, as well as ensuring program sustainability. The key themes are as follows: (1) population-level vulnerabilities to pneumonia, (2) disease burden, (3) policy processes, and (4) vaccine introduction optimization. Key findings are summarized in Table 2.

**Table 2.** Key findings from In-depth interviews.

Finding	Detail	Quote
Specific factors contribute to increased pneumonia vulnerability.	Climate change and maternal education were two of most cited factors. Environmental conditions such as flooding and changes in weather patterns were seen as having a negative impact on the burden of pneumonia. Poor maternal education was also associated with overlooking the danger signs of pneumonia and delaying care-seeking.	*“Remote areas are very hot, and climate is the most serious factor contributing to pneumococcal risk for children.” [SA]*
Gaps in country-level surveillance systems hinder efforts to study the potential impact of PCV introduction.	Participants described limited surveillance capacity as contributing to the lack of data on specific pneumonia burden. Strengthening surveillance systems was noted as being important to facilitate understanding of disease burden and vaccine impact. Public sector data sources could be supplemented with private hospital data.	*“We don’t have that kind of specific data (surveillance) for pneumonia in the country. We’re thinking of setting up a surveillance system so that prior to introduction we would have baseline data.” [SS]*
Despite concerns and potential barriers, there is strong commitment to develop and leverage decision-making processes to sustainably introduce PCV.	Participants described global advocacy, including a summit with leaders from multiple countries, as energizing efforts for PCV introduction. However, leaders expressed uncertainty about balancing multiple vaccine priorities, particularly in settings with less formalized technical advisory groups on immunization policy making. Further, the financial aspects of introduction, including logistics, were one of the largest considerations of vaccine introduction.	*“Who will pay for the vaccine? Partners? In any case, it is the state that must finance, and the modalities must be defined so that, once the vaccine is introduced, stock-outs can be avoided.” [GA]*
Key steps ahead of PCV introduction will bolster implementation and sustainability.	Implementation priorities to support the rapid and sustainable introduction of PCV include communication campaigns, enhancing cold chains, training for healthcare workers, and building on pandemic-related learnings.	*“They have introduced COVID-19 vaccine in a short time. This paves the way now for the Minister of Health to believe in themselves, and to have more experience in introducing new vaccines.” [SA]*

### Theme 1: population-level vulnerabilities to pneumonia

Participants highlighted the varying risks associated with increasing susceptibility to pneumonia.

#### Subtheme 1.1 – climatic risks

Participants emphasized widespread and heightened susceptibility to pneumonia and pneumococcal disease across the four countries, as many children are at high risk due to poverty and poor healthcare access. Participants indicated that specific populations had increased vulnerabilities due to environmental factors and climatic risks.

In Somalia, participants said that rural and nomadic populations exposed to climate disasters, as well as internally displaced people who lived in crowded dwellings such as temporary resettlement camps, were particularly vulnerable to pneumonia.

All three participants interviewed from South Sudan pointed toward flooding as one factor that increased population susceptibility to disease and also hindered vaccine rollout. One participant from a United Nations (UN) agency shared:

With the current flood and rains [due] … air shipment is [needed] to deliver vaccine. (SS-02)

In Guinea, participants discussed risks from household crowding, pollution, mining, and dust.

The incidence rate is highest due to these determinants … environmental factors, the crowdedness that some vulnerable communities live in and especially marginalized communities. (GA-01)

A participant speaking about Guinea from a UN agency also cited consistent pneumonia caseloads in hospitals year-round with regular spikes in cases between May and June, corresponding to the transition from Guinea’s ‘hot season’ to its ‘rainy season.’

#### Subtheme 1.2 – maternal education

Stakeholders from Chad, Guinea, and Somalia discussed poor maternal education and literacy as contributing to pneumonia susceptibility and to vaccine hesitancy.

In Somalia, lack of [maternal] education … malnutrition, and overcrowding contribute as risk factors of the battle of pneumonia disease. (SA-03)

Participants in these countries said many caregivers were uninformed about pneumonia danger signs and delayed care-seeking. As one policy maker said in Guinea:

For fear of spending too much on health care, they start with the traditional healer and providing first aid. [It] contributes to the worsening of the child’s health condition. (GA-01)

Vaccine hesitancy, also discussed below, was linked with poor levels of maternal education across several countries, while high education levels were connected to increased vaccine knowledge.

The [more] educated a mother is, the more she is aware of the benefits of vaccination. (CD-01)

### Theme 2: disease burden

Participants highlighted that there are currently gaps in country-level disease surveillance systems, which are needed to document the impact of PCV introduction.

#### Subtheme 2.1 – disease surveillance

All participants said that pneumonia was perceived as one of the leading causes of under-5 mortality. However, participants described limited capacity for bacterial surveillance, which contributed to a lack of data on the specific burden of pneumococcal disease.

In Somalia, participants stated that there were a dearth of laboratories with sufficient technical expertise and equipment to isolate bacteria, particularly fastidious organisms such as pneumococcus. However, this was not described as an obstacle for decision-making for new vaccine introductions, as global data on PCV impact as well as data from other countries in the region provide an understanding of the local disease burden and expected impact of preventative measures.

In the…[local] regions … like Kenya, we…have some stud[ies] which [are] not far [off in] the context of Somalia. Ethiopia is [also] a bordering country with Somalia. So those [countries give us] context [so] we can have some data [to] inform our context of Somalia. But we don’t have a really strong and integrated [surveillance] system in Somalia. (SA-04)

In Chad, a participant similarly discussed seeking to “replicate” the success PCV has had in other regional countries, with the caveat that they were concerned with “making sure that the children are sufficiently covered [with vaccination]” to achieve impact.

#### Subtheme 2.2 – surveillance strengthening for documentation

Participants from South Sudan stated that they aim to strengthen surveillance systems given the upcoming introduction of PCV, as administrative data can facilitate an understanding of vaccine impact.

Participants in other countries described using national health system information tools, specifically, increasing the use of District Health Information Software 2 (DHIS2) to track and report pneumonia cases and deaths.

Participants in Somalia and Guinea reported that although private facilities were not obligated to participate in official monitoring, data from the National Health Information System as well as the annual yearbook could be used to illustrate trends.

### Theme 3: policy process

Participants highlighted that, in spite of potential barriers impacting vaccine decision-making procsses, there is strong commitment to leverage existing support to facilitate the sustainable implementation of PCV.

#### Subtheme 3.1 – prioritization of vaccines

Acknowledging high childhood mortality from several vaccine-preventable diseases, participants indicated that other vaccines such as malaria or rotavirus vaccines are also being considered for introduction. Two participants from South Sudan stated that the country has prioritized introducing malaria vaccine and rotavirus over PCV.

We’re looking at malaria first based on the burden in South Sudan, then we look at rotavirus vaccine, and then at PCV. That’s how it has been spaced out within the country. (SS-01)

In Chad, some participants said the country had prioritized addressing malaria, which has long known to be a cause of significant morbidity and mortality, before they would be able to address pneumonia.

They [Chad’s government] want to reduce disease burden, especially for malaria, which has the highest prevalence. And then we can come to the next problems, which will be malnutrition and pneumonia. (CD-01)

Despite these multiple priorities, government sources seemed committed to rapidly introducing several vaccines. According to this leader, Chad was planning to catch-up on vaccine introductions to further improve child health.

We are a little bit late, [but now] we are introducing a lot of vaccines. There is the ROTA, then HPV that we have not yet introduced. (CD-04)

A participant in Chad also suggested that co-introduction of PCV with rotavirus vaccine could help the country more quickly facilitate national scale-up of both vaccines.

#### Subtheme 3.2 – financial considerations

Some participants shared that there were outstanding financial concerns to be addressed prior to the introduction of PCV. Co-financing, a requirement for countries to contribute to the cost of Gavi-supported vaccines by financing a small proportion of vaccine costs, was mentioned as a concern across Chad, Guinea, Somalia, and South Sudan.

The one big challenge we will be facing is co-financing. Because if we need to introduce any new vaccines including PCV vaccines, the country needs to contribute for the co-financing, which is $0.20 per dose. (SA-04)

Financing costs associated with operational activities, such as transportation and training, were also mentioned as a concern. One participant from Guinea said:

Who will pay for it? Partners? In any case, it is [the] state that must finance, [and] the modalities must be defined so that, once the vaccine is introduced, stock-outs can be avoided. (GA-01)

#### Subtheme 3.3 – decision making processes

Participants stated that vaccine recommendations were largely made by subcommittees within health ministries in consultation with technical specialists at WHO, UNICEF, and other public health agencies, rather than independent subject matter experts within a National Immunization Technical Advisory Group (NITAG).

However, participants in Guinea described an independent advisory group that made recommendations to its Ministry. In Chad, participants said a NITAG was in development, and that an Expanded Program on Immunization (EPI) technical support committee had previously made recommendations to the ministry.

In Somalia, participants explained that well-rounded technical committees and task forces were established to address vaccines, with representation from the MoH, WHO, UNICEF, and other agencies.

#### Subtheme 3.4 – advocacy

Despite these potential barriers, participants expressed strong political will to introduce PCV across all countries, with some of this motivation being enhanced by the second Global Forum on Childhood Pneumonia in March 2023.^3^

I believe really passionately that the vaccine will help to prevent childhood bacterial infections caused by pneumococci. (SA-02)

After this global conference, Somalia [will be] one of the countries that will start rolling out that [PCV] (SA-06)

A participant from Guinea shared that the Pneumonia Forum in Madrid offered an opportunity to gather support from the international community. They also appreciated the peer-learning opportunities that this Forum afforded.

We can benefit from the experience of countries that have already gone through this process to learn from the lessons already learned by others, so that we can move forward quickly and well. (GA-02)

### Theme 4: vaccine introduction optimization

Participants discussed priorities, including communication, infrastructure, cold chain and training, to support the rapid introduction and sustainability of PCV.

#### Subtheme 4.1 – communication

Most participants discussed varied and successful communication approaches that had been used to increase demand and counter hesitancy, particularly in the context of COVID-19 vaccines, and which could be leveraged to support PCV rollout.

In Somalia, the rollout of COVID-19 vaccines offered insights on effective vaccine communication strategies. Senior health and political leaders made supportive public statements to overcome hesitancy in advance of the COVID-19 vaccine rollout. Similarly, participants shared that the government planned to proactively release supportive statements by the president, religious leaders, regional governors, and community elders, ahead of a future PCV introduction. Partners in Somalia also used community outreach through social media, including push notifications to phones, as well as a network of social mobilizers that can be tapped to support future vaccine rollouts.

In Somalia, we have the Social Mobility Action Network. There is a platform of social mobilizers that were working on [vaccine] program[s], but now they are flexible to support other areas of health services, which included COVID-19 [vaccination campaigns]. That platform will help us to support the introduction of PCV because they are social mobilizers, they’ve been there for a long, and they have very good experience of the context of Somalia. (SA-04)

Participants in Guinea and South Sudan discussed community-based outreach used to strengthen vaccine uptake. In Chad, two participants discussed communication strategies extensively. COVID-19 misinformation, particularly on social media, significantly delayed vaccine scale-up, and participants felt that communication efforts were under-resourced in Chad. In Somalia, participants shared that an effective vaccine introduction campaign would need to involve national leaders, multiple languages, diverse religious leaders, traditional authorities, as well as community-based organizations that administer vaccines at the local level.

Participants from Chad and Guinea said local “sensitizers” or town criers – a traditional one-person messenger who moves through small villages announcing important news with a megaphone – have an important role in explaining the benefits of vaccines at the local level. However, not all participants discussed the need for communication, with one in Chad stating that logistical issues, such as stockouts and service availability, may impact vaccine uptake.

#### Subtheme 4.2 – infrastructure and capacity

Participants shared that poor access to health care, as a result of infrastructure and capacity, is common in these four countries. However, participants were optimistic that these existing infrastructure and capacity barriers could be navigated through effective partner engagement.

Participants across all countries said that programs suffered from limited transportation infrastructure, with one participant describing the sudden absence of road infrastructure upon leaving the capital of Chad. In South Sudan, limited road network and extensive flooding were cited as requiring expensive air transport for vaccine transport. Participants further reported that there was limited capacity across partners and the MoH to respond to competing priorities, including severe drought, measles outbreaks, and other emergencies straining government capacity.

Security was also noted as being a leading concern for health workers. In Somalia, participants shared that resurgent groups control a major part of Somalian territory in the south, and international NGOs and government offices encounter significant barriers in providing health services.

Participants shared that national and international partners played a significant role in the rollout of vaccines, particularly in countries experiencing major humanitarian emergencies. This partner supported allowed countries to fill existing capacity and infrastructure gaps. In Somalia, participants stated that partners support logistics, supply chain, and social and community mobilization. In other countries, partners were also reported to have provided health worker training and incentives, and assistance with managing health facilities. However, donor involvement was said to bring in an additional layer of complications across the countries, as there are different roles partners take on which may or may not be aligned with each other.

It’s [a] fragmented system – somewhere partners are working on some [work], others are doing their own work. (SA-03)

#### Subtheme 4.3 – cold chain

Given the widespread limitations on infrastructure, such as a limited or sporadic electricity, there are cold chain shortages across the four countries. However, COVID-19-related investments from partners were cited as alleviating the shortfall to some extent. In Somalia, one participant described:

Half of the facilities may not have a cold chain fridge, [but] we’re building the capacity of the cold chain at the facility level. SA-01

Participants in Chad and South Sudan recognized the additional capacity needed for PCV introduction, with one participant from the government saying, “we need to ensure that our cold chain can withstand these extra vaccines we’re bringing onto the system.”

#### Subtheme 4.4 – training

Participants agreed on the need for adequate training to prepare for PCV rollout, with some participants describing their concerns about the additional burden on health workers posed by vaccine introductions. In Somalia, a participant said that COVID-19 introduction-related trainings bolstered capacity of immunization-related health workers, but additional training would be needed for PCV.

In Chad, participants said that the health workforce is limited in quality and quantity. Participants in Somalia also discussed the need for strengthening vaccination platforms more generally, beyond health workers, encompassing specialists who can better support the regulatory system and marketing authorization.

#### Subtheme 4.5 – coverage

The struggle to increase low routine vaccine coverage, in conjunction with vaccine introduction, emerged unprompted in some, but not all, interviews. A ministry spokesperson in Guinea referred to “multiple epidemics” which created low coverage levels of other vaccines. From this participant, there was a sense that coverage rates for existing EPI vaccines should be higher before the country should initate a new vaccine introduction.

With Ebola, COVID-19, and others, there has been a slackening in immunization coverage. To introduce new vaccines, we first need to increase immunization coverage rates. (GA-02)

However, more participants emphasized the need to increase coverage of all vaccines in conjunction with PCV introduction to ensure vaccine effectiveness.

## Discussion

Chad, Guinea, Somalia, and South Sudan are all at pivotal moments in their journey to introduce PCV. There is widespread national commitment and external financial support to facilitate the rollout of PCV across the four countries.^25^ In examining the context for PCV introduction and implementation in four Gavi-eligible countries in Africa with fragile and humanitarian contexts, our findings suggest that it is critical to build on existing momentum to sustainably introduce PCV into routine immunization schedules; this introduction must be supplemented with concurrent strategies to increase community awareness, immunization coverage, and vaccine confidence. It is with an understanding of these concepts and country needs that partners can best support local leaders in their journey toward implementation.

The context for vaccine implementation in these countries – particularly given that these countries are ranked among the most vulnerable to climate change globally – requires researchers and implementation partners to consider climate-related increases in population vulnerability to infectious diseases along with greater program exposure to extreme weather events.^26,27^ Although climate change was not a topic about which interviewers initiated discussions, nearly all participants described environmental changes as complicating vaccine implementation. Current research forecasts that the burden of vaccine-preventable diseases will be influenced worldwide by climate change, for instance through increased humidity and rainfall which affects the burden of respiratory diseases.^28,29^ Further, climatic changes can impact prevention strategies such as vaccination, with vaccine storage, transportation, and routine immunization coverage being negatively affected. Our participants discussed heat waves as affecting the cold chain in Chad, and they described flooding as increasing the cost of vaccine transportation in South Sudan. A recent retrospective analysis of sub-Saharan African countries indicated that extreme weather consequences such as drought can negatively impact routine immunization coverage.^30^ Planned program enhancements supporting vaccine introductions, such as cold chain improvements, must address ongoing and future climatic events.

Maternal educational status is another factor increasing the vulnerability of the region’s children to pneumonia. Study participants described poorer caregiver awareness as increasing children’s susceptibility to severe disease, such as when parents overlook danger signs, delay care-seeking, or consult traditional practitioners. While the global community has targeted 90% coverage of appropriate case management by 2025, in Somalia and Chad, only 13% and 18% of under-5 children, respectively, with pneumonia (acute respiratory illness) symptoms are taken to consult with a health provider.^31,32^ Rates in Guinea and South Sudan are higher at 69% and 75%, respectively, although these are well below coverage targets.^33,34^ Further, low levels of maternal education were cited by participants in Chad, Guinea, and Somalia as contributing to vaccine hesitancy. Indeed, maternal education is key to drive higher levels of health service utilization, such as vaccine uptake.^35–37^ As such, efforts to increase caregivers’ awareness about pneumonia danger signs and associated health worker training and availability are needed alongside vaccine implementation. Although maternal education, and other barriers such as limited vaccine knowledge, are known to impact routine immunization uptake, there have been limited efforts to address these barriers. Often countries rely on immunization campaign materials from several decades ago although there is increasing recognition that the changing landscape of the community needs to be considered. Additionally, competing priorities result in countries attempting to simultaneously address urgent infrastructure and community needs. However, technical committees have been supporting countries with identifying ways to simultaneously address barriers to vaccine uptake. Lastly, country governments use their immunization advisory groups to work with technical committees and professional societies to increase public awareness and advocacy using evidence based approaches.

Introducing PCV into a country’s routine immunization program provides significant benefits for managing pneumococcal disease, leading to a reduction in mortality and morbidity from pneumonia, meningitis, and other infections. Yet participants in all four countries discussed the underlying challenge of suboptimal routine vaccine coverage, with low existing vaccine coverage rates (e.g., 42% coverage of DTP3 in Somalia) as having the potential to dampen the impact of a PCV vaccine rollout.^38^ While one participant said new vaccine introductions should follow efforts to increase coverage, leaders and partners may choose to emphasize that new vaccine introductions have the potential to generate new demand while simultaneously increasing uptake of existing vaccines within the national immunization program. Further, as interview participants said, the rapid introduction of COVID-19 vaccines has strengthened programs through health worker training and cold chain infrastructure. Building on this existing platform may facilitate the introduction and long-term sustainability of PCV programs.

Communication campaigns around new vaccines are a means for increasing demand, while refresher training for health workers associated with a new vaccine introduction should support improved service delivery. It is critical for the passion and urgency that participants expressed for PCV introduction to be translated into vocal, high-level political support during implementation. The COVID-19 pandemic brought about manifold lessons for demand generation, and new vaccine rollouts can build on existing community demand generation strategies, create locally tailored communication campaigns, and rely on locally embedded, trusted messengers, such as religious leaders and town criers, to drive uptake and acceptance in communities. Leaders can establish early warning systems to identify and respond to both misinformation and lower-than-expected uptake.^39,40^ Taken together, our findings highlight that the COVID-19 pandemic has strengthened both the supply and demand side of vaccinations.

Identifying context-specific determinants of implementation success will be valuable to building tailored introduction strategies for each country. To create long-term momentum, participants shared that it is essential to improve surveillance, health care infrastructure, and management capacity. Further, increasing the formality of the vaccine decision-making process through the strengthening of NITAGs may expedite the introduction of other WHO-recommended vaccines (e.g., RSV).^41^ These four countries are planning to introduce the rotavirus vaccine by 2025, and South Sudan, Chad, and Guinea have plans to introduce the malaria vaccine within the next two years. While none of these countries plan to introduce HPV vaccine in the short run, there are intentions to start applications in the coming years to facilitate introduction. This project was limited by several factors. Engaging participants for interviews was challenging as these countries were experiencing various emergencies in 2022 and 2023, including insurgencies, flooding, and disease outbreaks. Further, this research did not capture community perspectives, as our approach was focused on national technical and managerial leaders. Notwithstanding these limitations, by studying up and interviewing national and regional expert respondents, we focused on structural factors and felt needs related to vaccine introduction directly experienced by country stakeholders. In doing so, our inquiry sought to align with the decolonization movement in global health, which works to strengthen health systems from within, and allowing national leaders to guide discussions about what is needed to facilitate success.

Following the conduct of these interviews, Gavi announced their decision to provide additional support for countries in humanitarian crises at the Global Forum on Childhood Pneumonia in March 2023.^3,42^ With the help of Gavi, leaders across the four countries have made strong commitments to roll out the vaccine in 2024; leaders in these countries are now focusing on initiating introduction planning, selecting a vaccine schedule and product, and determining how to proceed programmatically. Two of these countries have successfully submitted formal applications to Gavi to secure support for new vaccine introduction, a critical step toward actual implementation of vaccine rollout.

Ensuring shared accountability between leaders and external partners for long-term program success will be essential. In such Sustainably introducing PCV in Chad, Guinea, Somalia, and South Sudan is a critical step in the global fight against pneumonia complex and sometimes fragmented settings, it is essential to nurture and maintain effective relationships between country leaders and global partners through participatory planning and implementation stages.

## Conclusion

Sustainably introducing PCV in Chad, Guinea, Somalia, and South Sudan is a critical step in the global fight against pneumonia. Expert advice from country leaders and partners indicate that in order to ensure the best outcomes in these countries, effective multi-sectoral collaborations are needed to support activities that address specific factors contributing to pneumonia susceptibility, strengthen country-level surveillance systems, and build on momentum to strengthen decision-making processes to bolster new vaccine implementation and sustainability.

## Supplementary Material

Supplemental Material
