# Supplementary material for: Introduction of the pneumococcal conjugate vaccine in humanitarian and fragile contexts: Perspectives from stakeholders in four African countries
Source: Hum Vaccin Immunother. 2024 Mar 5;20(1):2314828. doi: 10.1080/21645515.2024.2314828 (PMC10936592; doi:10.1080/21645515.2024.2314828)
Supplement: Supplemental Material [file KHVI_A_2314828_SM6769.pdf]

SUPPLEMENTAL MATERIAL

Appendix 1: Interview Guide

Note: Interviewers used subsets of these questions based on the interview.

Demographic Overview Questions

1. Please state your current job title, the name and location of the organization you work for:
2. How long have you been in this role?
3. What are your main responsibilities in this role?
4. Can you please describe your level of involvement and responsibilities with regards to: The National Immunization Program / Strategies for prevention, surveillance and treatment of childhood pneumonia

Perceptions of Pneumococcal disease burden

5. What is the burden of childhood pneumonia and pneumonia in your country?
6. What types of surveillance systems and data sources are generally used to track pneumococcal disease incidence and prevalence?

Perceptions of Pneumococcal disease susceptibility

7. Are there specific populations or places that are more at-risk for pneumococcal disease? What are contributors to the higher risk of children living in these places?
8. What social, environmental, economic, or other factors do you believe most contribute to pneumococcal disease burden in your country?

Perceptions of Health Systems Priorities

9. What are some of the government's top national priorities or targets to combat pneumonia, especially among children?
10. How would you describe the effectiveness of government efforts to control in your local health system?

Vaccine Decision-making Process

11. Can you describe the process by which decisions are made in [Country] around new vaccines?
12. Can we talk about the immunization strategic plan?
13. What are some of the opportunities and challenges for a government deciding to introduce PCV?
14. Who are the people involved in decision-making within the health ministry?

Vaccine Implementation

15. What support or resources would be required to overcome challenges with supply chains or other logistics?
16. What barriers or challenges may impact progress of PCV introduction and rollout?
17. How do you think PCV introduction may impact childhood health in your country?
18. What specific sub-national regions or populations of children do you believe should be prioritized during PCV introduction?

Vaccine uptake and acceptance

19. Please describe your experiences or takeaways from other vaccine introductions, such as COVID-19 vaccines, in your country. How would you suggest applying these lessons to the context of PCV introduction? / Which specific strategies would you recommend to maximize uptake for PCV rollout in children?
20. Are there any final thoughts or comments you would like to share?

21. Are there any other people who we should reach out to?
